# Circulating long non-coding RNAs HOTAIR, Linc-p21, GAS5 and XIST expression profiles in diffuse large B-cell lymphoma: association with R-CHOP responsiveness

**DOI:** 10.1038/s41598-021-81715-5

**Published:** 2021-01-22

**Authors:** Mahmoud A. Senousy, Aya M. El-Abd, Raafat R. Abdel-Malek, Sherine M. Rizk

**Affiliations:** 1 Department of Biochemistry, Faculty of Pharmacy, Cairo University, 23 Kasr Al-Ainy street, Cairo, 11562 Egypt; 2 General Administration of Clinical Trials, Central Administration of Biological and Innovative Products and Clinical Studies, Egyptian Drug Authority, 51 Wezaret El Zeraa Street, Agouza, Giza, Egypt; 3 Department of Clinical Oncology, Kasr Al-Ainy Centre of Clinical Oncology & Nuclear Medicine, Faculty of Medicine, Cairo University, Cairo, Egypt

**Keywords:** Biochemistry, Cancer, Genetics, Molecular biology, Biomarkers, Diseases, Molecular medicine, Oncology

## Abstract

The reliable identification of diffuse large B-cell lymphoma (DLBCL)-specific targets owns huge implications for its diagnosis and treatment. Long non-coding RNAs (lncRNAs) are implicated in DLBCL pathogenesis; however, circulating DLBCL-related lncRNAs are barely investigated. We investigated plasma lncRNAs; HOTAIR, Linc-p21, GAS5 and XIST as biomarkers for DLBCL diagnosis and responsiveness to R-CHOP therapy. Eighty-four DLBCL patients and thirty-three healthy controls were included. Only plasma HOTAIR, XIST and GAS5 were differentially expressed in DLBCL patients compared to controls. Pretreatment plasma HOTAIR was higher, whereas GAS5 was lower in non-responders than responders to R-CHOP. Plasma GAS5 demonstrated superior diagnostic accuracy (AUC = 0.97) whereas a panel of HOTAIR + GAS5 superiorly discriminated responders from non-responders by ROC analysis. In multivariate analysis, HOTAIR was an independent predictor of non-response. Among patients, plasma HOTAIR, Linc-p21 and XIST were correlated. Plasma GAS5 negatively correlated with International Prognostic Index, whereas HOTAIR positively correlated with performance status, denoting their prognostic potential. We constructed the lncRNAs-related protein–protein interaction networks linked to drug response via bioinformatics analysis. In conclusion, we introduce plasma HOTAIR, GAS5 and XIST as potential non-invasive diagnostic tools for DLBCL, and pretreatment HOTAIR and GAS5 as candidates for evaluating therapy response, with HOTAIR as a predictor of R-CHOP failure. We provide novel surrogates for future predictive studies in personalized medicine.

## Introduction

Diffuse large B-cell lymphoma (DLBCL) is the most common subtype of non-Hodgkin lymphoma (NHL), constituting up to 40% of all cases globally^1^. It is a cancer of B-cells that have been exposed to antigens, the annual incidence of which is rising year by year^1^. Notably, DLBCL is one of the most common NHL subtypes in North Africa and Middle East (49.4%) compared to North America (29.3%)^2^. Egypt exceptionally has high incidence of lymphoma and is claimed to have higher incidence of NHL among all hematopoietic cancers^3^.

DLBCL is a fast growing tumor that occurs in lymph nodes within the neck, armpit or groin area, but may appear elsewhere. It is diagnosed primarily by biopsy, complete blood count and computed tomography^4^. Largely, DLBCL is a rapidly progressive fatal malignancy that responds badly to existing treatment, with more than one-third of affected patients are resistant to various therapies^5^. The current standard initial therapy for DLBCL is a combination of Rituximab (CD20 antibody), cyclophosphamide, doxorubicin, vincristine, and prednisone (R-CHOP)^5^. Prognosis has improved significantly by adding Rituximab to the conventional therapy^6^, however approximately 30% to 50% of patients still respond badly to R-CHOP, depending on disease stage or prognostic index^7^. The most commonly used prognostic tool in DLBCL is the International Prognostic Index (IPI), which takes only into account clinical parameters such as age, clinical stage and performance status^8^. Recently, the deconvolution of the complex molecular genetics of DLBCL has unraveled key oncogenic pathways that improved the understanding of its biological diversity^5^. Thus, exploring novel genetic and/or epigenetic markers may be of clinical value for the diagnosis, prognosis, and therapy of DLBCL.

Long non-coding RNAs (lncRNAs), a class of ncRNAs longer than 200 nucleotides, are implicated in cancer initiation, development and progression through epigenetic regulation of multiple cellular paradigms^9^. Indeed, dysregulated lncRNAs act as oncogenes or tumor suppressors in diverse cancers including haematological malignancies, and have come out as interesting predictive biomarkers for diagnosis, prognosis, therapy responsiveness and also as therapeutic targets^9,10^. Intriguingly, the possible application of lncRNA-based therapies in clinical practice has attracted much attention in the last decade and many clinical trials are already started e.g., the DTA-H19 vector in bladder, ovarian, and pancreatic cancer^11,12^. Recently, the clinical application of lncRNAs in B-cell malignancies is increasingly evident based on their involvement in normal B-cell development as well as the pathogenesis of B-cell tumors^13,14^, however, the biological functions, expression pattern, and prognostic value of many lncRNAs in DLBCL are still largely unelucidated^13^. In addition, data about circulating DLBCL-related lncRNAs are scarce. Thus, profiling circulating lncRNAs may open a new avenue for non-invasive DLBCL diagnosis, treatment and prediction of its response to therapy.

HOX transcript antisense intergenic RNA (HOTAIR) is reported as an oncogenic lncRNA that promotes cell proliferation, tumor invasiveness and metastasis, and its overexpression is a marker of poor prognosis in various cancer types, including lymphoma^15^. LincRNA-p21 (Linc-p21), a p53-dependent lncRNA, is reported to be a tumor suppressor lncRNA in B-cell malignancies^16^. Growth arrest-specific transcript 5 (GAS5) is an another tumor suppressor lncRNA that regulates cell survival^17^, and was linked to B-cell lymphoma^18^. X-inactive-specific transcript (XIST) is a 17 kb lncRNA that sculpts the cis-inactivation of the over one thousand X-linked genes^19^. Indeed, ample evidence demonstrated aberrant XIST regulation in various cancers, including lymphoma and male testicular germ-cell tumors, where XIST hypomethylation was observed^20^.

In this study, we selected these 4 lncRNA candidates (HOTAIR, Linc-p21, GAS5 and XIST) based on the reported biological link with DLBCL or other lymphomatic cancers. HOTAIR and Linc-p21 have been shown to be dysregulated in DLBCL tissue and cell line samples^21,22^. GAS5 was reported to be deregulated in B-cell malignancies^18^ and in DLBCL in silico^23^. XIST was abnormally expressed in hematologic cancer^20,24^. However, the circulating expression profiles of these lncRNAs and their clinical relevance in diagnosis and response to therapy among DLBCL patients is still poorly investigated. This motivated us to investigate their plasma expression profiles and explore their potential as novel non-invasive diagnostic markers in DLBCL patients, their correlations with clinical data, and whether their pretreatment levels would predict patient responsiveness to R-CHOP therapy. Functional analysis was also performed using online databases and softwares to relate these lncRNAs to R-CHOP responsiveness.

## Results

### Patients’ characteristics and classifications

The clinicopathological characteristics of DLBCL and healthy controls are summarized in Table 1. 42% of patients presented with B-symptoms and 29% were having high serum lactate dehydrogenase (LDH) levels. 49% of patients had Ann Arbor stages III-IV. 36% of patients scored IPI 3–4 with poor prognosis.

**Table 1:** Demographic and clinical characteristics of DLBCL patients and healthy controls.

Parameter	Patients	Control	*P* value
	n = 84	n = 33	
**Age > 18, mean ± SD**	50 ± 13.30	47.21 ± 10.52	0.28
**Range, years**	21–79	30–70	
**Sex, n (%)**			
Male	37 (44%)	18 (54.5%)	0.41
Female	47 (56%)	15 (45.5%)	
**Ann Arbor stage, n (%)**			
I	14 (17%)	–	
II	29 (34%)		
III	19 (23%)		
IV	22 (26%)		
**ECOG PS, n (%)**			
0	13 (15.5%)		
1	39 (46%)		
2	13 (15.5%)		
3	16 (19%)		
4	3 (4%)		
**IPI, n (%)**			
0	17 (20%)	–	
1	21 (25%)		
2	16 (19%)		
3	16 (19%)		
4	14 (17%)		
**Extranodal lymph nodes, n (%)**			
≥ 2	37 (44%)	–	
< 2	47 (56%)		
**B-Symptoms, n (%)**			
Present	35 (42%)	–	
Absent	49 (58%)		
**Bulk, n (%)**			
≥ 5 cm	54 (64%)	–	
< 5 cm	30 (36%)		
**LDH level, n (%)**			
normal	60 (71%)	–	
high	24 (29%)		
**Family history, n (%)**			
+ ve	13 (15%)	–	
−ve	71 (85%)		

Data are expressed as mean ± SD or number (percentage).

*IPI* International Prognostic Index, *LDH* lactate dehydrogenase, *PS* performance status.

**Figure 1:**
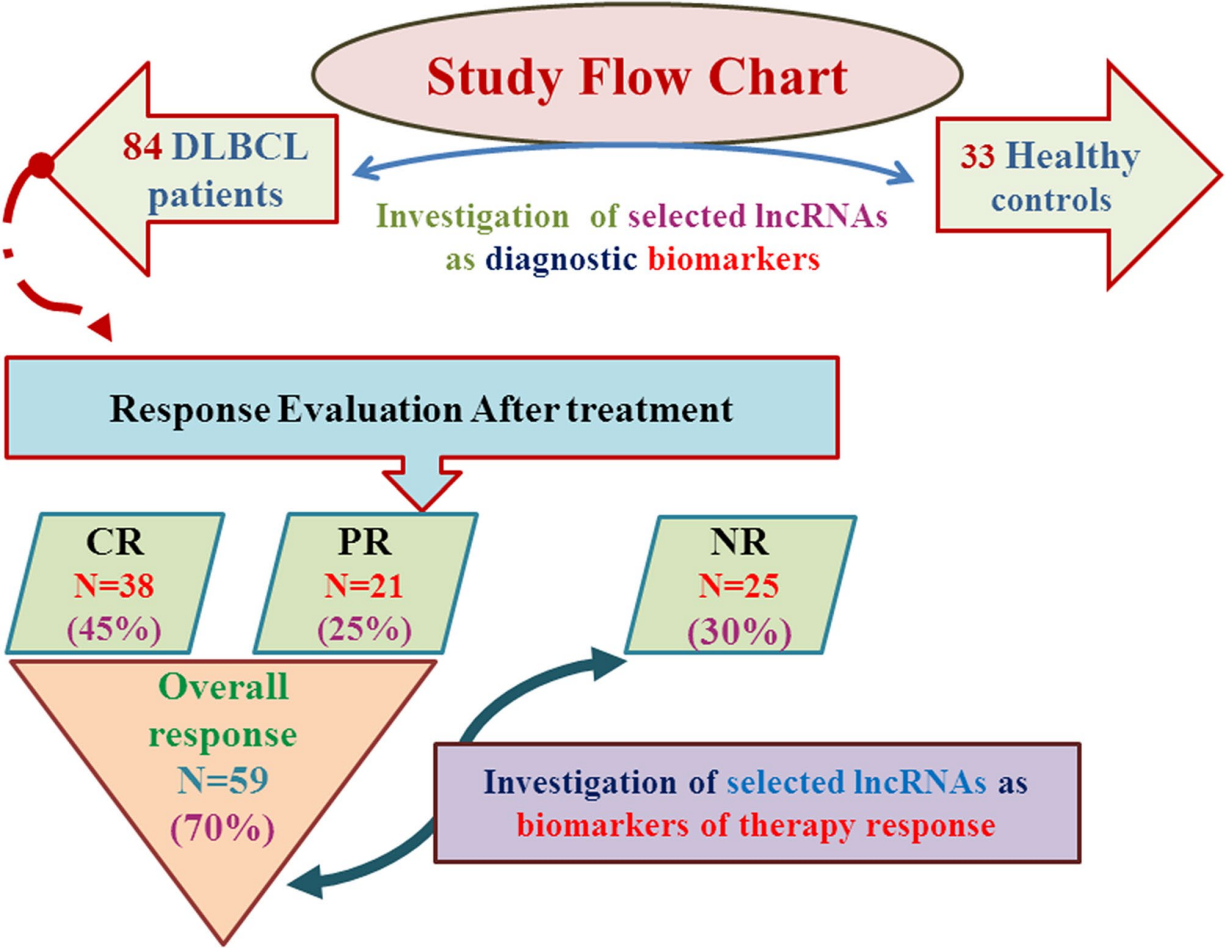
Flow chart of study design, patient classification, treatment response and analysis data sets. *CR* complete response, *PR* partial response, *NR* non-responders, *N* number.

Patients were classified based on their end of study treatment response as shown in Fig. 1. After completion of the R-CHOP treatment cycles, DLBCL patients (n = 84) were classified according to response evaluation criteria into complete responders (CR), n = 38; partial responders (PR), n = 21; and non-responders (NR), n = 25. Thus, the overall responders (CR + PR) were 59/84 (70%), while the NR patients were 25/84 (30%). Comparison of the clinicopathological data of overall responder and NR groups revealed statistically significant higher IPI scores in NRs compared to the overall responders (*P* = 0.014). NRs were of higher age than overall responders (*P* = 0.01). Other clinicopathological data showed no significant difference between the two groups (Table 2).

**Table 2:** Clinical data of overall responders and non-responders to R-CHOP therapy.

Parameter	Overall responders	Non-responders	*P* value
	n = 59	n = 25	
Age ≤ 60	50 (85%)	14 (56%)	0.01*
> 60	9 (15%)	11 (44%)	
**Sex, n (%)**			
Male	29 (49%)	8 (32%)	0.16
Female	30 (51%)	17 (68%)	
**Ann Arbor stage, n (%)**			
I/II	33 (56%)	10 (40%)	0.23
III/IV	26 (44%)	15 (60%)	
**ECOG PS**			
0–2	48 (81%)	17 (68%)	0.25
3–4	11 (19%)	8 (32%)	
**IPI, n (%)**			
0–2	43 (73%)	11 (44%)	0.014*
3–4	16 (27%)	14 (56%)	
**Extranodal lymph nodes, n (%)**			
< 2	35 (59%)	12 (48%)	0.35
≥ 2	24 (41%)	13 (52%)	
**B-symptoms, n (%)**			
Present	25 (42%)	10 (40%)	1
Absent	34 (58%)	15 (60%)	
**Bulk, n (%)**			
≥ 5 cm	41 (69%)	13 (52%)	0.14
< 5 cm	18 (31%)	12 (48%)	
**LDH level, n (%)**			
Normal	41 (69%)	19 (76%)	0.61
High	18 (31%)	6 (24%)	
**Family history**			
+ ve	11 (18%)	2 (8%)	0.33
−ve	48 (81%)	23 (92%)	

Data are expressed as number (percentage).

*IPI* International Prognostic Index, *LDH* lactate dehydrogenase, *PS* performance status.

*Indicates statistical significance *P* < 0.05.

### Plasma lncRNAs levels in DLBCL patients

**Figure 2:**
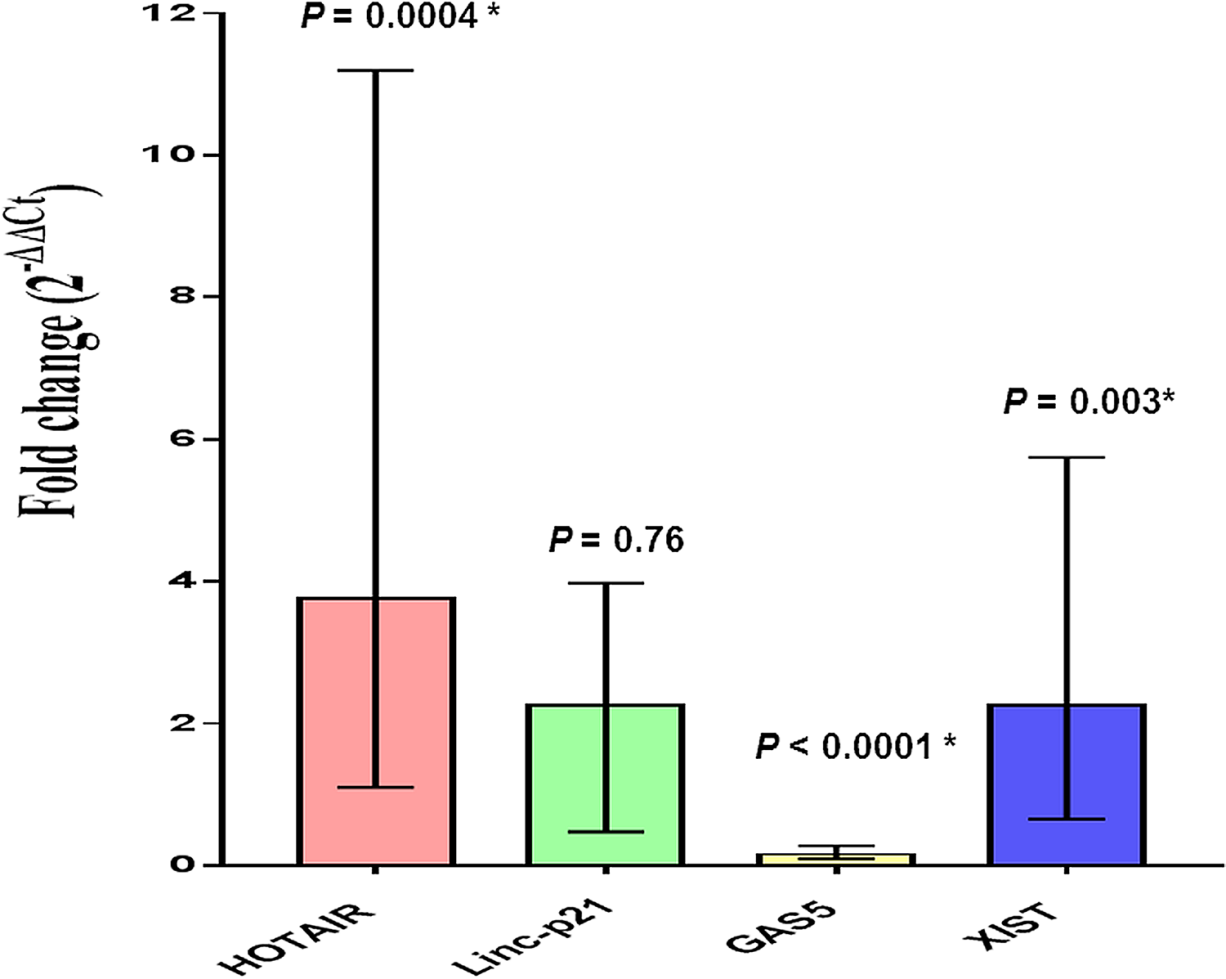
Plasma fold change levels of studied lncRNAs in DLBCL patients compared to healthy controls. Data are presented by median interquartile range. DLBCL, n = 84, controls, n = 33. *Indicates statistical significance (*P* < 0.05).

All studied lncRNAs were expressed in control plasma with varying levels (Supplementary Fig. S1). HOTAIR and XIST levels were significantly upregulated with a median fold change = 3.77, *P* = 0.0004 and 2.265, *P* = 0.003, respectively, whereas GAS5 expression was significantly downregulated with a median fold change = 0.159 (*P* < 0.0001) in the overall DLBCL patients compared to the control group. On the other hand, Linc-p21 expression was not statistically significant between the two groups (*P* = 0.76) (Fig. 2).

### Pretreatment plasma lncRNAs levels and responsiveness to R-CHOP therapy

**Figure 3:**
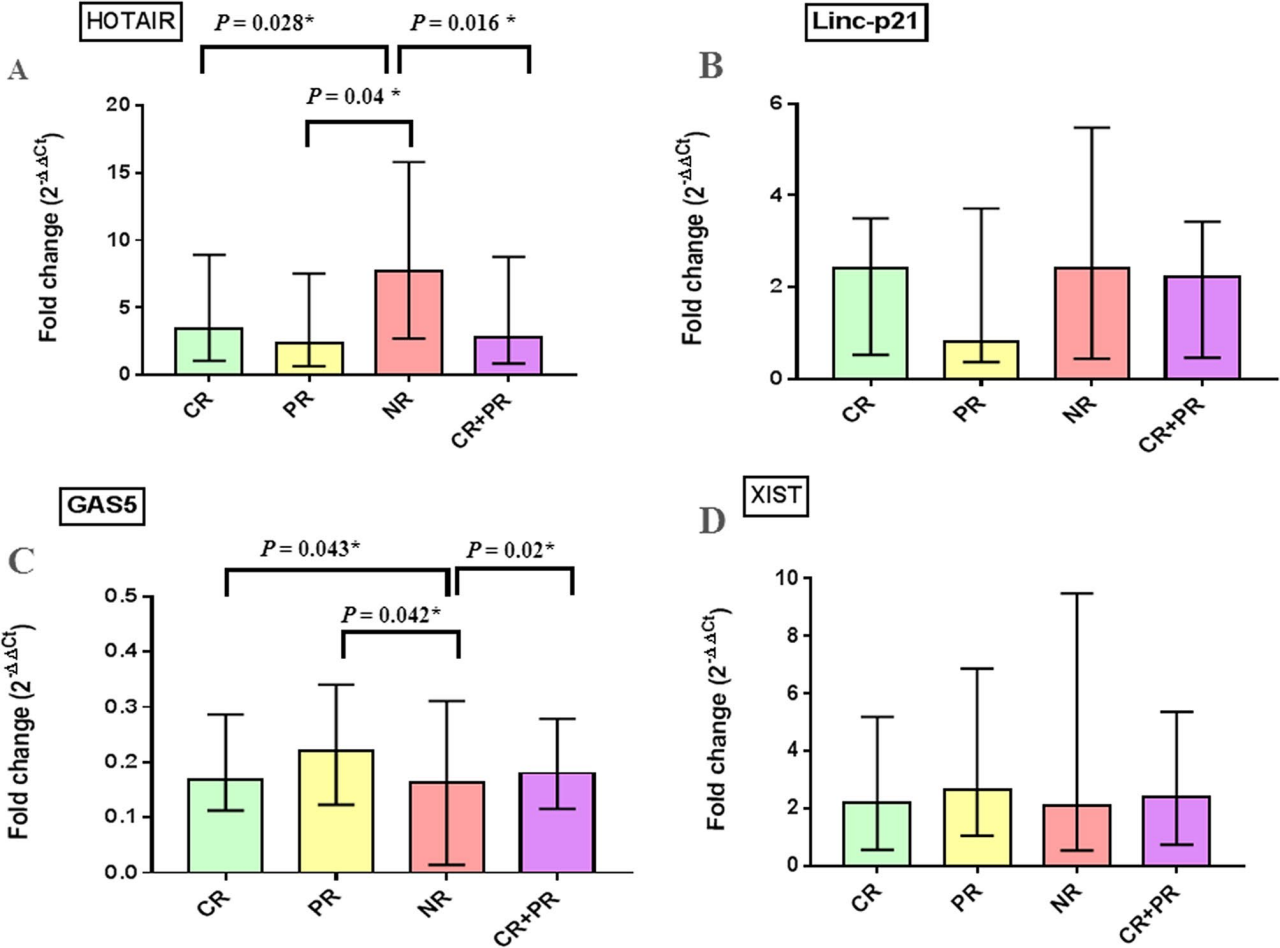
Baseline plasma fold change of studied lncRNAs in relation to treatment response. Data are presented by median interquartile range. Complete response (CR), n = 38, partial response (PR), n = 21 and non-responders (NR), n = 25, overall responders (CR + PR), n = 59. *Indicates statistical significance (*P* < 0.05).

Baseline plasma lncRNAs levels in DLBCL patients were analyzed in relation to response outcome (Fig. 3). Pretreatment levels of plasma HOTAIR were significantly higher in NR than those in CR or PR groups (*P* = 0.028, *P* = 0.04 respectively). Indeed, further analysis revealed that baseline plasma HOTAIR levels were higher in NR than overall responders (CR + PR) (*P* = 0.016). On the other hand, GAS5 levels were significantly higher in CR or PR than NR groups (*P* = 0.043, *P* = 0.042, respectively). Further analysis showed that the levels of GAS5 in plasma of overall responders were significantly higher than those in NRs (*P* = 0.02). Comparisons of the pretreatment HOTAIR and GAS5 levels between CR vs PR + NR revealed no statistical difference (*P* > 0.05). On the other hand, pretreatment plasma Linc-p21 and XIST levels were not statistically different at all comparisons (*P* > 0.05) (Fig. 3).

### Diagnostic and prognostic potentials of studied plasma lncRNAs

**Figure 4:**
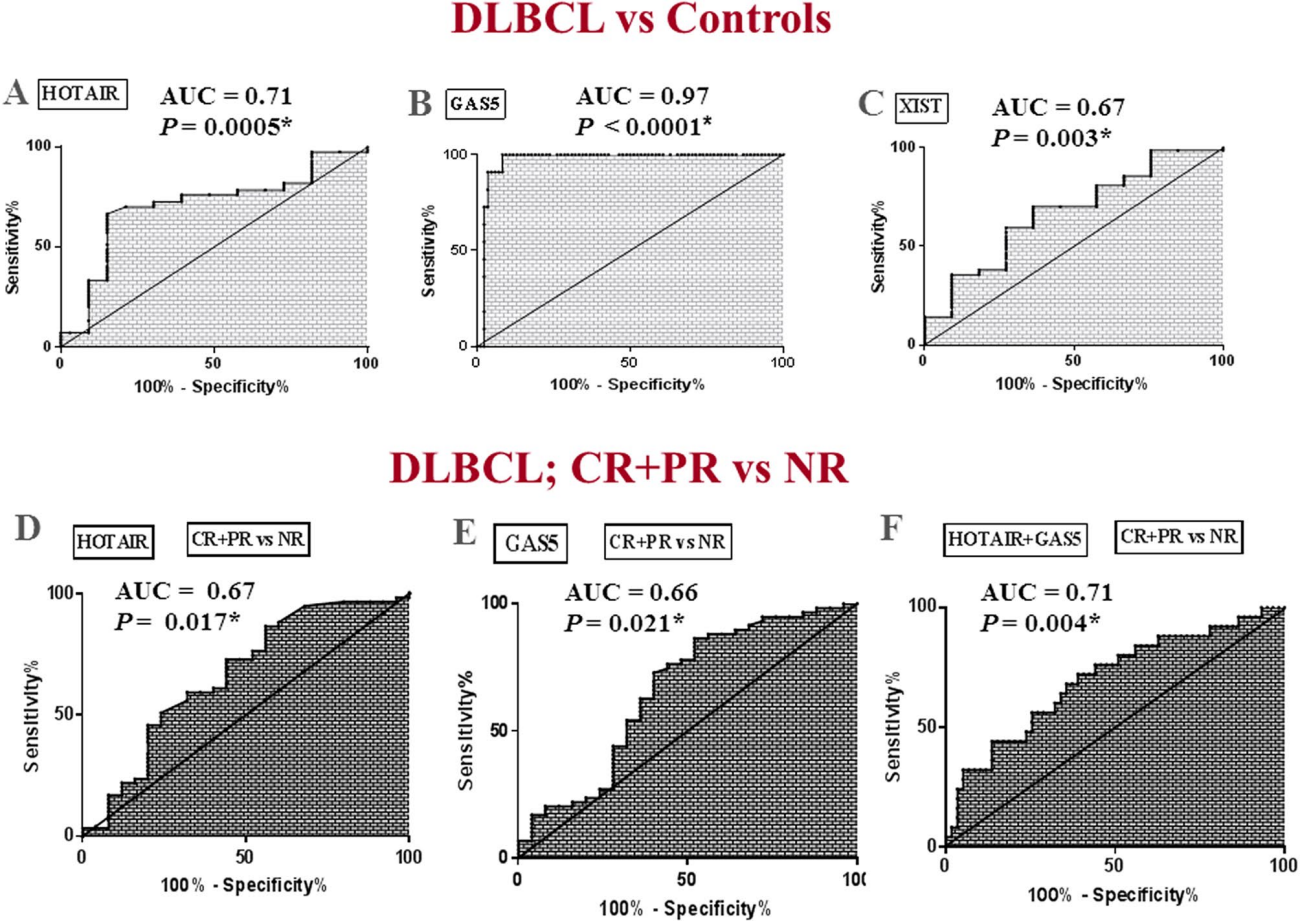
Plasma lncRNAs as biomarkers of DLBCL diagnosis and therapy outcome. (**A–C**) ROC curve analysis of plasma (**A**) HOTAIR, (**B**) GAS5 and (**C**) XIST as diagnostic biomarkers differentiating DLBCL patients (n = 84) from healthy controls (n = 33). (**D–F**) ROC analysis of plasma (**D**) HOTAIR and (**E**) GAS5, (**F**) HOTAIR + GAS5 to differentiate overall responders (n = 59) from NR (n = 25) among DLBCL patients.

Receiver-operating-characteristic (ROC) analysis was performed to explore the clinical value of HOTAIR, GAS5 and XIST in the diagnosis of DLBCL (Fig. 4A–C). Cut-off points were determined such that they maximized the sum of sensitivity and specificity. Results showed an AUC of 0.71 for HOTAIR (95%CI = 0.602–0.814, *P* = 0.0005), 0.97 for GAS5 (95%CI = 0.935–1.002, *P* < 0.0001), and 0.67 for XIST (95%CI = 0.566–0.782, *P* = 0.003). The optimal sensitivity and specificity to differentiate DLBCL from healthy controls were 72.62% and 69.7%, respectively at a cutoff fold change > 1.34 for HOTAIR, 91.67% and 100%, respectively at a cutoff fold change < 0.45 for GAS5, 70.24% and 63.64%, respectively at a cutoff fold change > 1.05 for XIST. These results demonstrate the impact of these lncRNAs as diagnostic biomarkers in DLBCL. Comparison of the ROC curve results suggested that plasma GAS5 performed much better (AUC = 0.97) than HOTAIR and XIST (AUC = 0.71, 0.67, respectively, differences = 0.26, 0.3, *P* < 0.0001, respectively).

The prognostic significance of plasma HOTAIR and GAS5 which were differentially expressed in overall responders and NR groups were evaluated using a ROC curve (Fig. 4D,E). Results revealed that baseline plasma HOTAIR and GAS5 levels discriminated patients with different treatment outcome among DLBCL patients with AUC of 0.67 (95%CI = 0.5302 to 0.802, *P* = 0.017), and 0.66 (95%CI = 0.521–0.798, *P* = 0.021), respectively. The optimal sensitivity and specificity to discriminate overall responders from NR patients were 72.8% and 56%, respectively at a cutoff fold change < 2.37 for HOTAIR and 67.8% and 60%, respectively at a cutoff fold change > 0.13 for GAS5. Combination analysis of HOTAIR + GAS5 (Fig. 4F) revealed that a panel of baseline plasma HOTAIR plus GAS5 levels discriminated patients with different treatment outcome among DLBCL patients with AUC = 0.71 (95%CI = 0.574 to 0.824, *P* = 0.004), with optimal sensitivity and specificity of 72% and 61.02%, respectively. These results demonstrate the impact of these lncRNAs as biomarkers of therapy outcome in DLBCL.

### Correlation between lncRNAs levels and clinical data

**Figure 5:**
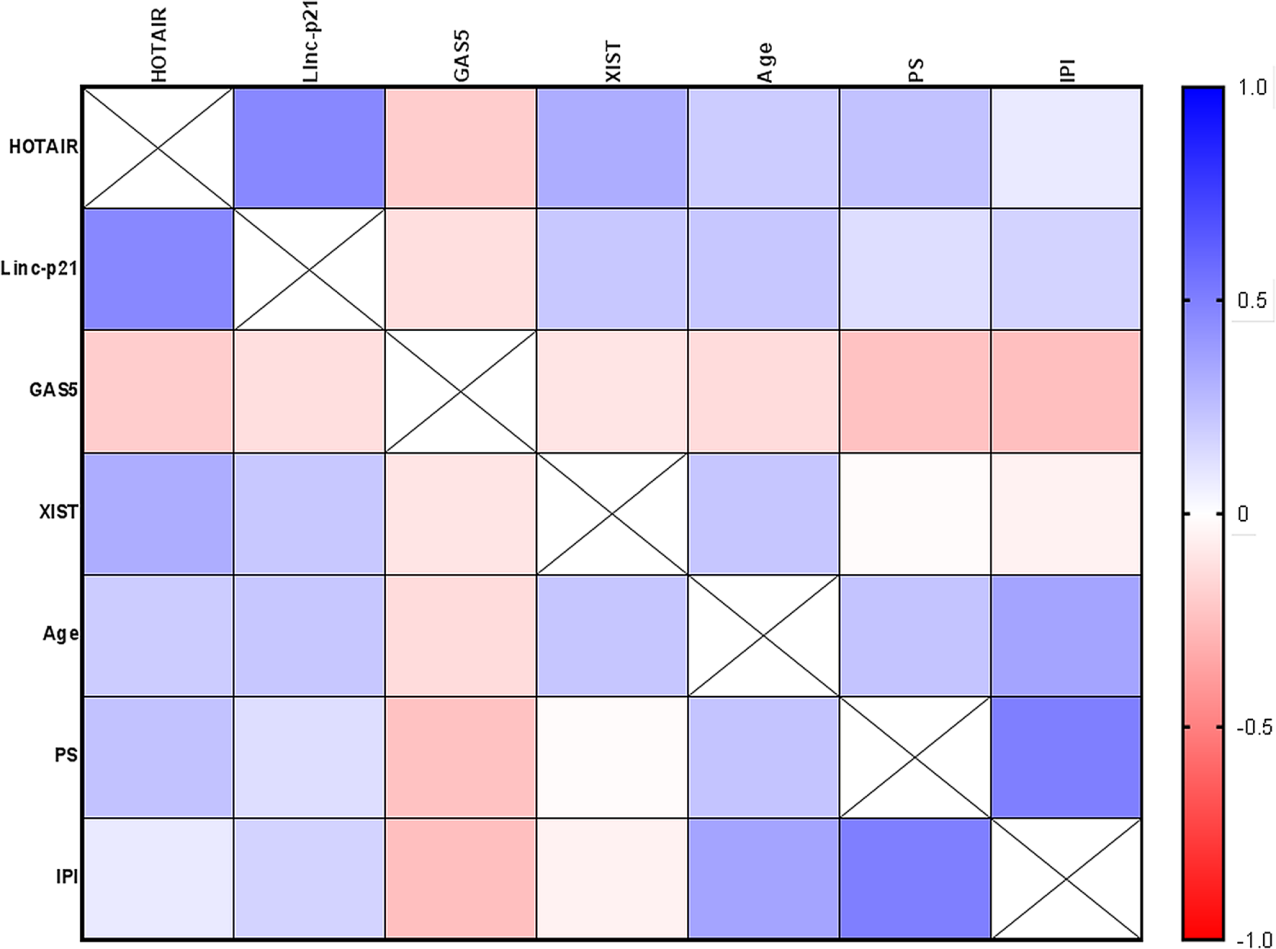
Correlation between plasma lncRNAs levels with each other and with clinical data. A correlation map with a blue-red scale. The blue color corresponds to a correlation close to 1 and the red color corresponds to a correlation close to − 1. Correlations are made by Spearman correlation. *IPI* International Prognostic Index, *PS* performance status.

We further examined correlations between studied plasma lncRNAs with each other and with clinical data in the overall studied DLBCL patients (Fig. 5 and Supplementary Table S1). HOTAIR levels were positively correlated with Linc-p21 (r = 0.46, *P* < 0.0001), XIST (r = 0.32, *P* = 0.003) levels and PS (r = 0.24, *P* = 0.029). Linc-p21 levels were positively correlated with XIST levels (r = 0.215, *P* = 0.049) and patient age (r = 0.217, *P* = 0.047). Plasma GAS5 levels were negatively correlated with IPI (r = -0.251, *P* = 0.022) and PS (r = -0.243, *P* = 0.026). Finally, XIST levels were positively correlated with patient age (r = 0.22, *P* = 0.044).

### Prediction of DLBCL diagnosis and therapy outcome

Univariate and multivariate logistic regression analyses were performed to identify predictors of the risk of DLBCL diagnosis and its treatment outcome. Neither studied lncRNA was able to predict the risk of being diagnosed with DLBCL (DLBCL vs healthy controls) in the univariate analysis (Supplementary Table S2). Interestingly, HOTAIR was selected as significant negative predictor of overall response to therapy in DLBCL patients (*P* = 0.008) in the univariate analysis. GAS5 demonstrated marginal association (*P* = 0.045) in the univariate analysis. In the multivariate analysis, HOTAIR was the final independent negative predictor of overall response. In other words, HOTAIR was an independent predictor of non-response. Results were adjusted with age, sex, family history and IPI score as confounders (Table 3). These results suggested that HOTAIR may offer potential as biomarker for R-CHOP response evaluation in DLBCL patients.

**Table 3:** Plasma lncRNAs as predictors of overall response to R-CHOP in DLBCL patients.

Variable	Univariate analysis					Multivariate analysis				
	B	S.E	*P*	OR	95%CI	B	S.E	*P*^a^	OR	95%CI
HOTAIR	− 0.51	0.196	0.008*	0.60	0.407–0.877	− 0.44	0.206	0.032*	0.64	0.43–0.964
GAS5	7.06	3.130	0.045*	68.74	1.005–151	4.23	3.930	0.281	68.84	0.311–152.3

HOTAIR and GAS5 were included in a multivariate analysis with age, sex, family history and IPI score as covariates. − 2 Log likelihood of the best model, *P* < 0.0001.

^a^Adjusted with age, sex, family history and IPI score.

*Indicates statistical significance (*P* < 0.05).

### Results of functional analysis in relation to therapy response

**Figure 6:**
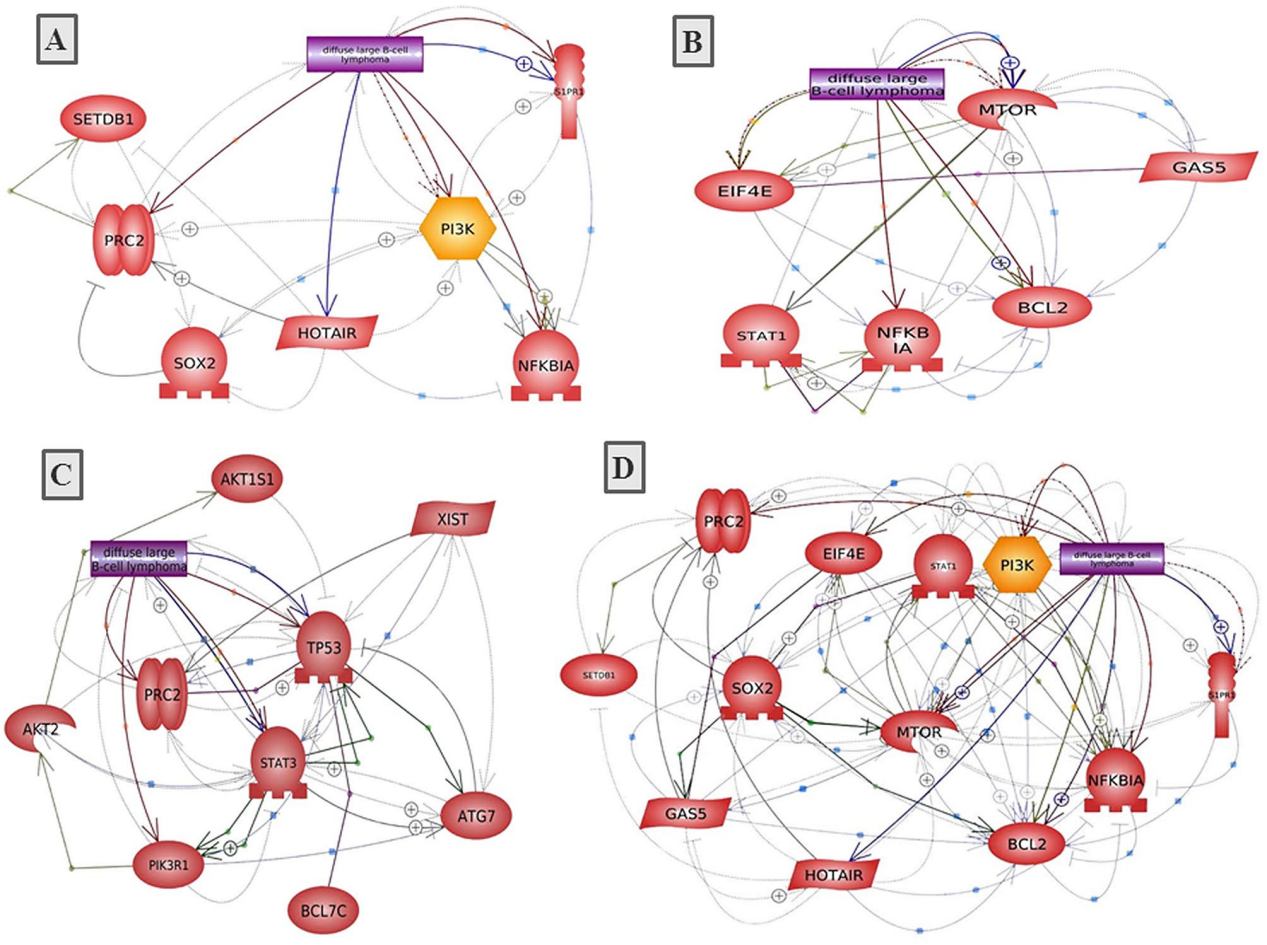
Construction of lncRNA-related PPI networks linked to drug responsiveness in DLBCL. (**A**) HOTAIR, (**B**) GAS5, (**C**) XIST, (**D**) HOTAIR + GAS5. Pathway Studio online software was used.

We carried out a functional analysis of DLBCL-associated lncRNAs in our study in relation to drug response. Of the large number of lncRNA-RNA interactions found in the starBase platform (http://starbase.sysu.edu.cn/) for HOTAIR, GAS5 and XIST, we selected the protein-coding genes, which were then filtered according to their possible biological relation to drug responsiveness using molecular annotation (MAS) system (http://bioinfo.capitalbio.com/mas3/). Finally, PI3K, PRC2, SOX2, IkBa, SETDB1 and S1PR1 were selected for HOTAIR; eIF4E, mTOR, STAT1, NFκBIA and BCL2 for GAS5; and TP53, STAT3, ATG7, PRC2, BCL7C, BCL79L, PIK3R1, AKT2, AKT1S1 for XIST (Table 4). To further identify the role of these lncRNAs-related genes, we analyzed the lncRNA-related protein–protein interactions (PPI) as well as the biological processes and KEGG pathways of the PPI network using the STRING online software. *P* values and the results of Gene ontology (GO) and KEGG pathway analyses for each lncRNA-related PPI are listed in Table 4. The lncRNA-related PPI network construction is visualized in Fig. 6.

**Table 4:** Bioinformatics analysis of the lncRNAs-related genes and protein–protein interactions linked to drug responsiveness.

lncRNA	lncRNA-related genes	PPI *P* value	Gene ontology for PPI network		KEGG pathway analysis for PPI network	
			Biological process	Strength (FDR)	Pathway	Strength (FDR)
HOTAIR	PI3K, PRC2, SOX2, IkBa, SETDB1,S1PR1	0.019*	Cell chemotaxis	1.55 (0.0209)	B-cell receptor signaling	1.96 (0.0105)
			Angiogenesis	1.34 (0.0398)	Chronic myeloid leukemia	1.93 (0.0105)
			Negative regulation of cellular process	0.56 (0.0368)	T-cell receptor signaling	1.82 (0.0105)
			Multicellular organism development	0.54 (0.0436)	TNF signaling	1.78 (0.0105)
			Signal transduction	0.54 (0.0436)	Apoptosis	1.68 (0.0105)
			Cellular response to stimulus	0.5 (0.0194)	Pathways in cancer	1.1 (0.0371)
GAS5	eIF4E, mTOR, STAT1, NFKBIA, BCL2	0.019*	Negative regulation of autophagy	2.03 (0.0055)	EGFR tyrosine kinase inhibitor resistance	2.0 (0.0012)
			TNF-mediated signaling	1.99 (0.0056)	NF-κB signaling	1.93 (0.0013)
			Positive regulation of cell growth	1.69 (0.0103)	Jak-STAT signaling	1.87 (0.00011)
			Immune response-activating signal transduction	1.37 (0.0203)	Apoptosis	1.67 (0.0015)
			Response to drug	1.24 (0.0027)	MicroRNAs in cancer	1.72 (0.0017)
			Negative regulation of cell differentiation	1.24 (0.0082)	Pathways in cancer	1.48 (0.00011)
			Negative regulation of cell cycle	1.18 (0.0364)	PI3K-AKT signaling	1.35 (0.0069)
			Positive regulation of cell Population proliferation	1.13 (0.0113)		
			Apoptotic process	1.11 (0.0115)		
XIST	TP53, STAT3, ATG7, PRC2, BCL7C, BCL79L, PIK3R1, AKT2, AKT1S1	0.0233*	Regulation of cell cycle arrest	1.64 (0.0102)	Acute myeloid leukemia	2.05 (0.00002)
			Lymphocyte differentiation	1.34 (0.0227)	Platinum drug resistance	2.02 (0.000018)
			Regulation of gene expression, epigenetic	1.29 (0.0258)	Chronic myeloid leukemia	1.98 (0.00002)
			Response to antibiotic	1.21 (0.0342)	B-cell receptor signaling	1.84 (0.00076)
			Cellular response to drug	1.2 (0.0347)	Apoptosis	1.74 (0.00006)
			Negative regulation of cell death	1.11 (0.0011)	mTOR signaling	1.7 (0.00007)
			Negative regulation of apoptotic process	1.06 (0.0045)	T-cell receptor signaling	1.69 (0.0013)
			Immune effector process	0.9 (0.0282)	Jak-STAT signaling	1.66 (0.00007)
			Cell differentiation	0.55 (0.0326)	TNF signaling	1.66 (0.0015)
					PI3K-AKT signaling	1.32 (0.00065)
					Pathways in cancer	1.28 (0.000085)
					MAPK signaling	1.22 (0.0084)

The PPI and functional enrichment analysis for the PPI were conducted using SPRING software. PPI, protein–protein interactions; FDR, false discovery rate.

*Indicates statistical significance (*P* < 0.05).

## Discussion

The pathogenesis of DLBCL involve multi-step and heterogeneous processes with different genetic and epigenetic changes, and that high epigenomic heterogeneity correlated with a higher relapse rate and poor outcome^25,26^. The lack of clear symptoms and early detection makes it difficult to diagnose at an early stage, leading to poor prognosis. Existing molecular prognostic markers of DLBCL include MYC, P53, BCL2, and Ki-67. However, they have several limitations. MYC and P53 mutations are found in only 10% and 15–30% of DLBCL patients, respectively^27^. BCL2 is upregulated in 40–60% of patients and is associated with worse outcomes only in certain subtypes of DLBCL, while data about Ki-67 were controversial^27^, necessitating the identification of new predictive markers. Recently, expectations have been raised regarding the potential role of lncRNAs as predictive markers^28–30^ and as potential mediators of resistance to cancer therapy^31,32^, however, these studies were carried out on tumor tissue samples and/or cell lines.

Herein, we found that plasma HOTAIR, XIST and GAS5 were differentially expressed in DLBCL patients indicating their involvement in the pathogenesis of DLBCL. To the best of our knowledge, we are the first to provide evidence about XIST expression in DLBCL and its diagnostic and prognostic significance. In addition, we demonstrated that plasma levels of HOTAIR, GAS5 and XIST showed a discriminative ability for DLBCL, suggesting them as surrogate non-invasive biomarkers of DLBCL diagnosis, with GAS5 was of superior diagnostic performance. We also recorded that baseline plasma HOTAIR and GAS5 were associated with prognosis and therapy outcome, with HOTAIR demonstrated predictive ability for R-CHOP failure. We also constructed the HOTAIR- and GAS5-related PPI networks to explore their role in drug response in DLBCL. Our results introduce GAS5 and HOTAIR as novel candidates for future large scale predictive studies in personalized medicine.

First-line early R-CHOP failure in DLBCL still represents a dramatic situation in routine clinical practice^33^. Among patients for whom R-CHOP therapy fails, 20% suffer from primary refractory disease (progress during or right after treatment) whereas 30% relapse after achieving complete remission^34^. Herein, we found that R-CHOP failure was 30%, that is in marginal compliance with the reported 30–50% failure^7,33,34^. Our findings could be due to the short-term follow up study design aiming at early detection of NR patients for shifting them to another treatment. Actually, a high rate of relapse usually appears during longer follow up periods^34^. Our results showed higher IPI scores in NR relative to overall responders, confirming that patients with a more advanced disease state have poorer prognosis and are more liable to R-CHOP failure.

Several mechanisms of resistance may account for refractoriness to R-CHOP in DLBCL. The majority of DLBCL patients present a double rearrangement of MYC and BCL2 genes called double-hit lymphoma (DHL), a chromosomal breakpoint, affecting the MYC/8q24 locus in combination with another recurrent breakpoint, usually BCL2 [t(14;18)(q32;q21)], although BCL6/MYC-positive DHLs or BCL2/BCL6/MYC-positive triple-hit lymphomas (THLs) may also be observed. All studies that focused on DHLs or THLs concluded that the patients outcomes were poor, with R-CHOP probably not being the best therapeutic option^35,36^. Furthermore, TP53, FOXO1, MLL3, CCND3, NFKBIZ, and STAT6 were identified as top candidate genes for therapeutic resistance in DLBCL^37^. In addition, lncRNAs play a crucial role in the chromosome breaks involved in typical gene rearrangements in hematologic malignancies^38^, and indirectly affect drug resistance through regulating the expression of some intermediate regulatory factors^31,32^.

Only a limited number of studies have examined circulatory levels of lncRNAs in B-cell malignancies^39,40^. Herein, the observed upregulation of plasma HOTAIR in DLBCL patients agreed with the previously reported overexpression in DLBCL tissues^21,41^ and cell lines^21^. Our recorded correlation between HOTAIR and performance status links this lncRNA to DLBCL prognosis. Similarly, HOTAIR upregulation in DLBCL tumor tissues was correlated with clinical stage, B symptoms, IPI scores and tumor volumes, and predicted poor prognosis and poor survival rates in DLBCL patients^21^. In addition, we highlighted an association of plasma HOTAIR with non-response to R-CHOP. Similarly, HOTAIR upregulation was associated with resistance to different chemotherapeutic drugs in non-small cell lung cancer (NSCLC), breast, and ovarian cancers via activating multiple oncogenic events^32^. In fact, HOTAIR upregulation seems to be a common event underlying cancer progression and resistance to therapy via a key pro-oncogenic role^15^, however, its role in inducing drug resistance in DLBCL was not fully clear.

Using a bioinformatics approach, we identified a HOTAIR-related PPI network linked to drug resistance in DLBCL, including PI3K, PRC2, SOX2, IkBa, SETDB1 and S1PR1. This PPI was enriched in cell chemotaxis and angiogenesis process and in B-cell and T-cell receptors signaling, TNF signaling and apoptotic pathways. To put this in context, HOTAIR promotes cell growth and inhibits apoptosis by regulating H3K27me3 and activating the PI3K/AKT/NF-κB pathway^42^, which is considered a checkpoint for R-CHOP resistance in DLBCL; PI3K/AKT inhibition was found to reverse R-CHOP resistance by destabilizing SOX2 in DLBCL^43^. HOTAIR also regulates chromatin remodeling in DLBCL via recruiting of polycomb repressive complex 2 (PRC2) proteins and inducing silencing of target genes through H3K27 trimethylation^41^. HOTAIR was hypothesized to inhibit IkBa (an inhibitor of NF-kB), and then activates c-MYC expression, which in turn induces HOTAIR expression through SETDB1/STAT3 signaling pathway involved in cisplatin-resistant ovarian cancer^44^. NF-κB mutations and high S1PR1 and S1PR1/pSTAT3 expression were known pathways contributed to increase relapse in DLBCL^34^. HOTAIR may also contribute to drug resistance through regulation of miR-130a that was associated with higher risk of R-CHOP failure in DLBCL^34^. HOTAIR is a direct target of c-MYC through interaction with putative c-MYC target response element in the upstream region of HOTAIR by harboring a miR-130a binding site^44^. Taken together, these results conceptualize the critical role of HOTAIR in drug resistance for R-CHOP in DLBCL, and provide HOTAIR as a therapeutic target.

Our study also demonstrated plasma GAS5 downregulation by a median 6.29 fold in DLBCL patients. Similar findings have been reported in B-cell neoplasm such as multiple myloma^39^. GAS5 was also reported to be abnormally expressed in DLBCL in an in silico analysis^23^. We recorded an inverse correlation of GAS5 and IPI, suggesting that low plasma GAS5 levels are incorporated in the pathogenesis and development of DLBCL and may correspond to the degree of prognosis. Indeed, patients with low GAS5 expression exhibited shorter overall survival than those with higher expression and GAS5 expression was an independent indicator of colorectal cancer (CRC) prognosis^45^. Additionally, we showed that higher baseline GAS5 was associated with good response to R-CHOP. To further analyze the role of GAS5 in drug response, we identified a GAS5-related PPI network which included eIF4E, mTOR, STAT1, NFKBIA and BCL2. This PPI was enriched in negative regulation of autophagy, cell cycle and cell differentiation, immune response activation, promotion of apoptosis and cell response to drugs via several pathways, including EGFR, NF-κB, JAK/STAT, and PI3K/AKT/mTOR signaling pathways. This agrees with previous reports that GAS5 is required for the inhibition of human T cell proliferation by mTOR antagonists^46^. In fact, GAS5 influences cell survival rate by activating the apoptotic machinery. Indeed, overexpression of GAS5 promoted apoptosis by decreasing the expression of the anti-apoptosis protein BCL-2 and inhibited tumor resistance to therapy in bladder and cervical cancers^32^. In addition, GAS5 binds directly to eIF4E, a key factor of translation initiation complex, then negatively affects the c-MYC protein through lncRNA–mRNA interaction, denoting that GAS5 overexpression promotes favorable response by indirectly regulating c-MYC^47^.

We found an upregulation of plasma XIST level in DLBCL patients. Similarly, serum XIST was found to be upregulated in NSCLC patients^48^. Mechanistically, XIST binds PRC2 and propagate epigenetic silencing of an individual X chromosome^49^. The transcription factor, Yin Yang 1 has also been reported to interact with and relocate XIST, to the inactivated X-chromosome in activated B-cells, thereby changing the X-linked gene regulation in these cells compared to antigen naïve B-cells^50^.

Although XIST expression was linked to therapeutic response in CRC, NSCLC, and ovarian cancer^24,51,52^, we failed to find a correlation between XIST and R-CHOP therapy responsiveness in DLBCL. This may be due to different cancer type, different therapy, regimen and population. Indeed, previous studies were heterogenous regarding the role of XIST in therapy responsiveness. While XIST was associated with doxorubicin resistance in CRC cells^24^ and cisplatin resistance in NSCLC^51^, it was correlated with Taxol sensitivity in ovarian cell lines^52^. To further unravel the role of XIST in drug resistance, our bioinformatics analysis included XIST. An XIST-related PPI network included TP53, STAT3, ATG7, PRC2, BCL7C, BCL79L, PIK3R1, AKT2 and AKT1S1 and was enriched in regulation of cell cycle arrest, lymphocyte differentiation, response to antibiotic, cellular response to drug, immune effector process and negative regulation of apoptotic process. KEGG pathway analysis revealed involvement in B-cell and T-cell receptors signaling, JAK/STAT, TNF, and PI3K/AKT, mTOR, MAPK signaling pathways. Further studies are needed to explore the exact mechanism of XIST in R-CHOP therapy at the cellular level.

Our finding that Linc-p21 expression was not changed in DLBCL patients compared to controls contrasts Linc-p21 downregulation in DLBCL tumor tissues^22^ and in circulation of acute lymphoblastic leukemia patients^39^. The reported low abundance of Linc-p21 may be due to the lack of functional tumor suppressor p53 protein which is located on chromosome 17. Deletions of chromosome 17 are frequent events in B-cell malignancies^25^. p53 may be also inactivated by the BCL6 gene during the genesis of lymphoma^25^.

We observed a positive correlation of Linc-p21 with HOTAIR and XIST levels, suggesting their concomitant expression in DLBCL to orchestrate several pathologic events and co-regulatory networks. Intriguingly, tissue Linc-p21 was correlated with clinicopathological data and considered an indicator of favorable clinical outcome and survival rates in DLBCL patients^22^. Linc-p21 was shown to impair tumerigenesis in DLBCL patients with an R-CHOP regimen^22^. However, we failed to find this relation. Discrepant results may be due to different type of sample (plasma vs tissue), sample size, sample collection and processing and the normalization method.

Few lncRNAs have been reported to be dysregulated in DLBCL tissue samples and cell lines and their abnormal expression levels were associated with poor prognosis^21,22,28–30,53,54^, with little were correlated with response to therapy^22,53^. Our study improves over previous studies in that it introduces circulating lncRNAs as novel complementary biomarkers in DLBCL diagnosis, prognosis and prediction of patient responsiveness to R-CHOP therapy. Moreover, our data emphasize HOTAIR as a predictor of R-CHOP failure and GAS5 as a good indicator for R-CHOP overall response in DLBCL and highlight some target genes relating them to drug resistance, which need further validation. Our findings provide useful rationale for personalizing anti-cancer therapy.

Yet, there are few limitations in the current study, involving relatively small sample size and missing further validation. Our study is also missing a survival analysis due to the one-end point study design which focused in response to therapy. Therefore, future aspects should be assigned for validation and further clarification of the biological function of circulating lncRNAs in DLBCL.

## Conclusion

Plasma HOTAIR, GAS5 and XIST could serve as novel non-invasive diagnostic biomarkers for DLBCL. Plasma GAS5 demonstrated superior diagnostic accuracy and was a candidate for DLBCL prognosis. Baseline plasma HOTAIR and GAS5 levels were associated with responsiveness of DLBCL patients to standard R-CHOP therapy, with pretreatment HOTAIR was able to predict treatment failure. Our data could have impact in personalized medicine where predicting positive response could save time, costs, and side effects. Our results also pave the way for identification and development of new lncRNA-diagnostic and therapeutic targets that could be translated into clinical practice.

## Subjects and methods

### Patients

Overall, 84 Egyptian patients with DLBCL and 33 age- and sex-matched healthy controls were included in this prospective study. The demographic data of patients and controls are listed in Table 1. DLBCL is diagnosed primarily by biopsy, complete blood count and computed tomography. Pathologically diagnosed DLBCL patients were admitted at the outpatient’s clinic of Kasr Al-Ainy Centre of Clinical Oncology & Nuclear Medicine (NEMROCK), Faculty of Medicine, Cairo University, Cairo, Egypt to receive standard treatment regimen; a combination of Rituximab (R) and conventional chemotherapy. The anthracycline-containing (R-CHOP) regimen included Rituximab 375 mg/m^2^ on day 1, cyclophosphamide 750 mg/m^2^ on day 2, doxorubicin 50 mg/m^2^ on day 2, vincristine 1.4 mg/m^2^ (up to a maximal dose of 2 mg) on day 2, and prednisone 40 mg/m^2^ for 5 days. DLBCL patients received R-CHOP therapy for total 6 treatment cycles (1 cycle every 21 days)^4^. Cut off assessment for treatment response was done one month after finishing the 6 cycles of treatment. Overall, the study period including patient enrollment and follow up was from January 2017 to August 2018.

All patients were subjected to full history taking and clinical examination. The inclusion criteria included patients with age > 18 years, gender of both sex, pathologically diagnosed as DLBCL patients and fit to receive chemotherapy. Patients who received previous treatment with Rituximab were excluded.

Written informed consents were obtained from all participants. The study protocol and informed consent were approved by the ethics committee of the Faculty of Pharmacy, Cairo University (No. BC1927) and complied with the good clinical practice (GCP) and Declaration of Helsinki guidelines.

### Definition of treatment response

After treatment cycles, patients were revaluated by using Fluorodeoxyglucose-Positron Emission Tomography/Computed Tomography (FDG-PET/CT) which is the recommended standard for post-treatment assessment in DLBCL. All recruited patients were successfully followed up till the end of study. At the end of therapy, patients were divided into three groups; responded to treatment, partially responded and non-responded according to the response evaluation (Fig. 1). Response was defined by comparing the residual uptake with the tumor uptake in baseline scan using FDG-PET/CT. Complete metabolic response (CR) is defined when no residual uptake exists, partial metabolic response (PR) when the uptake has decreased, and no metabolic response (NR) when it has not changed or progressive metabolic disease (PMD) when it has increased^55^. Overall response was defined as CR + PR.

### Data collection

Clinical, laboratory and pathology data as well as imaging studies were collected by reviewing the medical records of each participant. Clinical data included age, gender, lymphoma stage (Ann Arbor stage), Eastern Cooperative Oncology Group (ECOG) performance status, and the presence of B cell-related symptoms. IPI was calculated using age, clinical stage and performance status^4,8^. Laboratory data included a complete blood count and serum LDH level.

### Samples collection and plasma preparation

Blood samples were taken at baseline before starting therapy. After the patient had been diagnosed with DLBCL and complied with the inclusion criteria, a blood sample was withdrawn at the morning on the day of starting treatment (day 1 of cycle 1 of R-CHOP for each patient) according to the R-CHOP protocol. Samples were processed within 30 min to 2 h after collection. For RNA analysis, we used platelet-poor plasma to exclude cellular nucleic acids. Cell and cell components-free plasma was prepared from up to 5 ml whole blood collected on EDTA-coated tubes via a two-step centrifugation protocol (2000×*g* for 10 min at 4 °C and 12,000×*g* for 10 min at 4 °C) to thoroughly remove cellular nucleic acids. After separation, plasma was transferred to nuclease-free tubes in aliquots and stored at -80 °C until RNA extraction. Samples with hemolysis were excluded.

### LncRNAs assay

Total RNA was drawn out from 200 μl plasma using the QIAzol reagent by miRNeasy Mini Extraction kit (Qiagen, Valencia, CA, USA) according to the manufacturer’s instructions. The concentration and purity of RNA were determined using NanoDrop 2000 Spectrophotometer (Thermo Fisher Scientific, USA), and samples with a A260/A280 ratio between 1.8 and 2.0 were used in reverse transcription (RT). RNA samples were stored in nuclease-free tubes and stored at − 80 °C till further analysis.

RT was carried out on 100 ng of total RNA in a final volume of 20 μl RT reactions (incubated for 10 min at 25 °C then for 30 min at 50 °C and finally for 5 min at 85 °C) using the Maxima First Strand cDNA Synthesis kit (Thermo Fisher Scientific, USA) according to the manufacturer’s instructions. cDNA samples were stored in nuclease-free tubes and stored at − 80 °C till further analysis.

Expression of lncRNAs were evaluated by quantitative PCR analysis conducted using customized primers and Maxima SYBR Green qPCR Master Mix (Thermo Fisher Scientific, USA) according to the manufacturer’s protocol. We used GAPDH as the endogenous control to normalize lncRNAs. GAPDH was reported to be stably expressed in plasma and was previously selected as an internal control in plasma to normalize lncRNAs^56,57^. In addition, GAPDH level was not affected by age, sex and pathology in human plasma^57^, and was regarded as an ideal internal control for plasma assays^56,57^. The primers sequences were as follows: 5′-GGTAGAAAAAGCAACCACGAAGC-3′ (forward) and 5′-ACATAAA-CCTCTGTCTGTGAGTGCC-3′ (reverse) for HOTAIR; 5′-GGGTGGCTCACTCTTCTGGC-3′ (forward) and 5′-TGGCCTTGCCCGGGCTTGTC-3′ (reverse) for Linc-p21; 5′-GTGTGGCTCTGG-ATAGCAC-3′ (forward) and 5′-ACCCAAGCAAGTCATCCATG-3′ (reverse) for GAS5; 5′-GCATAACTCGGCTTAGGGCT-3′ (forward) and 5′- TCCTCTGCCTGACCTGCTAT-3′ (reverse) for XIST; 5′-CCCTTCATTGACCTCAACTA-3′ (forward) and 5′-TGGAAGATGGTGATGGGATT -3′ (reverse) for GAPDH.

For real-time PCR analysis of each lncRNA, 3 μl of RT products was mixed with 7.5 μl RNase-free water, 12.5 μl Maxima SYBR Green qPCR Master Mix and 1 μl forward primer and 1 μl reverse primer. The real-time amplification was performed using 25 μl reaction mixtures using the Stratagene Mx3005P QPCR System (Agilent Technologies, Germany) with the following conditions: 95 °C for 10 min, followed by 40 cycles at 95 °C for 15 s and 60 °C for 60 s.

ΔCT was calculated by subtracting the Ct values of GAPDH from the Ct values of the target lncRNAs. lncRNAs expression relative to internal control was calculated by 2^−ΔCt^. Fold change relative to healthy controls was calculated using 2^−ΔΔCT^ method.

### Functional analysis of lncRNAs-related genes in relation to therapy response

The starBase platform (http://starbase.sysu.edu.cn/) was used to check the candidate lncRNAs-RNA interactions. The output was filtered by selecting protein-coding genes. Then data were analyzed using MAS system provided by CapitalBio company (Molecule Annotation System, http://bioinfo.capitalbio.com/mas3/) to determine the biological roles of these lncRNA-related protein-coding genes. The genes most related to DLBCL, drug responsiveness and cancer therapy in terms of biological process, molecular function, and KEGG pathway analysis were finally selected. The cutoff *P* value was 0.05. STRING online software was used to analyze the interaction relationships between the proteins encoded by the selected lncRNA-related genes (protein–protein interactions, PPI). Functional enrichments; GO and KEGG pathway analysis were also conducted to determine the involvement of each lncRNA-related PPI in different biological pathways using STRING online software. The lncRNA-related PPI network was visualized using the Pathway Studio Online Software.

### Statistical analysis

Values are expressed as mean ± SD, median interquartile range, or number (percentage) when appropriate. According to data normality, comparison of independent samples from two groups was performed using Student’s t test or the Mann–Whitney *U*-test when appropriate. Because data were not normally distributed according to Shapiro–Wilk and Kolmogorov–Smirnov normality tests, comparisons of lncRNAs levels were performed by applying Mann–Whitney *U*-test or Kruskal–Wallis test followed by Dunn’s test for multiple comparisons when appropriate, and the expression levels of lncRNAs were presented in median interquartile range. To compare categorical data, Fischer exact test was performed. ROC analysis was performed to assess the diagnostic and prognostic accuracy and the area under the curve (AUC) was calculated. Logistic regression analysis was performed to identify predictors of DLBCL diagnosis and treatment outcome. Data that were significant according to the univariate analysis were then entered into multivariate analysis to determine the best model for identifying the final independent predictor variables, adjusted by confounders. Associations between parameters were determined by Spearman correlation. We considered *P* to be significant at < 0.05 with a 95% confidence interval (CI). All statistical analyses were performed using GraphPad Prism 7.0 and 8 (GraphPad Software, CA, USA) and DTREG software (Tennessee, USA).

### Ethics approval

Written informed consents were obtained from all participants. The study protocol and informed consent were approved by the ethics committee of the Faculty of Pharmacy, Cairo University (No. BC1927) and complied with the good clinical practice (GCP) and Declaration of Helsinki guidelines.

## Supplementary Information

Supplementary Information.

## Data Availability

All data generated or analyzed during this study are included in this article.

## Abbreviations

ATG7: Autophagy related 7
AKT1S1: AKT1 substrate 1 (proline-rich)
AKT2: AKT serine/threonine kinase 2
AUC: Area under the curve
BCL2: B-cell lymphoma 2
BCL7C: B-Cell CLL/Lymphoma 7 Protein Family Member C
CR: Complete response
CRC: Colorectal cancer
DHL: Double-hit lymphoma
DLBCL: Diffuse large B-cell lymphoma
ECOG: Eastern Cooperative Oncology Group
eIF4E: Eukaryotic translation initiation factor 4E
FDG-PET/CT: Fluorodeoxyglucose-Positron Emission Tomography/Computed Tomography
FDR: False discovery rate
GAS5: Growth arrest-specific transcript 5
GO: Gene Ontology
HOTAIR: HOX transcript antisense intergenic RNA
KEGG: Kyoto Encyclopedia of Genes & Genomes
lincRNAs: Long intergenic RNA
IPI: International Prognostic Index
LDH: Lactate dehydrogenase
lncRNAs: Long non-coding RNAs
mTOR: Mammalian target of rapamycin
NFKBIA: Nuclear Factor Kappa-B Inhibitor Alpha
NSCLC: Non-small cell lung cancer
NR: Non-responders
PR: Partial response
PRC2: Polycomb repressive complex 2
PS: Performance status
PPI: Protein–protein interaction
R-CHOP: Rituximab, cyclophosphamide, doxorubicin, vincristine, and prednisone
PI3K: Phosphoinositide-3-kinase
PIK3R1: Phosphoinositide-3-Kinase Regulatory Subunit 1
ROC: Receiver-operating-characteristic
SOX2: SRY-box 2
SETDB1: SET Domain Bifurcated 1
S1PR1: Sphingosine-1-Phosphate Receptor 1
STAT: Signal transducer and activator of transcription
THL: Triple-hit lymphomas
TP53: Tumor protein p53
XIST: X-inactive-specific transcript
