# Supplementary material for: Circulating long non-coding RNAs HOTAIR, Linc-p21, GAS5 and XIST expression profiles in diffuse large B-cell lymphoma: association with R-CHOP responsiveness
Source: Sci Rep. 2021 Jan 22;11:2095. doi: 10.1038/s41598-021-81715-5 (PMC7822898; doi:10.1038/s41598-021-81715-5)
Supplement: Supplementary file 1 — Supplementary Information. [file 41598_2021_81715_MOESM1_ESM.docx]

***Circulating long non-coding RNAs HOTAIR, Linc-p21, GAS5 and XIST expression profiles in diffuse large B-cell lymphoma: association with R-CHOP responsiveness***

**Mahmoud A. Senousy^a^, Aya M. El- Abd^b*^, Raafat R. Abdel-Malek^c^, Sherine M. Rizk^a*^**

^a^Department of Biochemistry, Faculty of Pharmacy, Cairo University, Cairo, Egypt.

^b^General Administration of Clinical Trials, Central Administration of Biological and Innovative Products and Clinical Studies, Egyptian Drug Authority, Giza, Egypt.

^c^Department of Clinical Oncology,Kasr Al-Aini Centre of Clinical Oncology & Nuclear Medicine, Kasr Al-Aini Faculty Of Medicine, Cairo University, Cairo, Egypt.

^*^Corresponding authors:

**Aya M. El-Abd**

Postal address: 51 wezaret El Zeraa Street, Agouza, Giza, Egypt.

Mobile number: +20 1002776474

E-mail: aya.elabd@gmail.com

[aya.elabd@norcb.gov.eg](mailto:aya.elabd@norcb.gov.eg)

**Sherine M. Rizk**

Postal address: 23 Kasr Al-Aini street, Cairo, Egypt

Postal code: 11562

Mobile number: +201223302925

E-mail: [sherine.abdelaziz@pharma.cu.edu.eg](mailto:sherine.abdelaziz@pharma.cu.edu.eg)

**Supplementary Figure S1.**

**
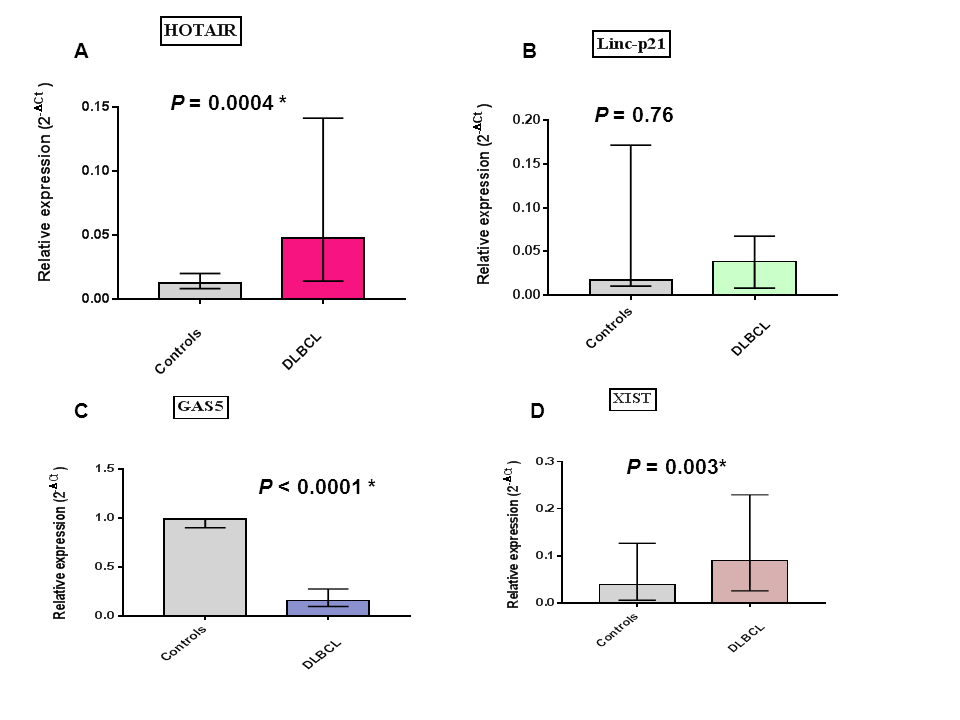
**

**Plasma expression levels of lncRNAs in DLBCL patients versus healthy control.** Data were represented by median interquartile range in DLBCL, n=84 vs healthy controls, n=33. Data were analyzed by Mann-Whitney U test. *indicates statistical significance (*P*<0.05).

**Supplementary Table S1. Correlation between LncRNAs with each other and with clinical data**

|  |  | HOTAIR | Linc-p21 | GAS5 | XIST | Family History | SA | Age | LDH | Ann Arbor stage | PS | Extranodal Status | IPI | Bulk | B- symptoms |
| --- | --- | --- | --- | --- | --- | --- | --- | --- | --- | --- | --- | --- | --- | --- | --- |
| HOTAIR | r |  | 0.46343 | -0.1995 | 0.32153 | 0.12152 | -0.0233 | 0.19676 | -0.0414 | -0.0253 | 0.23862 | -0.047 | 0.08013 | -0.1348 | 0.01145 |
|  | *P* |  | **9E-06** | 0.0688 | **0.00286** | 0.27083 | 0.83466 | 0.07282 | 0.7088 | 0.81964 | **0.02882** | 0.67126 | 0.47148 | 0.22161 | 0.91763 |
| Linc-p21 | r | 0.46343 |  | -0.1263 | 0.21469 | 0.05771 | -0.0314 | 0.21667 | -0.0242 | 0.03426 | 0.12754 | 0.10535 | 0.17196 | -0.0928 | 0.00398 |
|  | *P* | **9E-06** |  | 0.25217 | **0.04986** | 0.60208 | 0.77822 | **0.04775** | 0.82726 | 0.75702 | 0.24763 | 0.3402 | 0.12007 | 0.40136 | 0.9713 |
| GAS5 | r | -0.1995 | -0.1263 |  | -0.1038 | -0.1045 | 0.07986 | -0.1404 | -0.0021 | 0.02688 | -0.2426 | -0.0851 | -0.251 | 0.02459 | -0.1897 |
|  | *P* | 0.0688 | 0.25217 |  | 0.34719 | 0.34401 | 0.47296 | 0.20286 | 0.98453 | 0.80826 | **0.0262** | 0.44174 | **0.02209** | 0.82427 | 0.0839 |
| XIST | r | 0.32153 | 0.21469 | -0.1038 |  | -0.0346 | -0.1695 | 0.22001 | -0.1171 | -0.1691 | -0.0178 | 0.02275 | -0.0525 | -0.1091 | -0.0767 |
|  | *P* | **0.00286** | **0.04986** | 0.34719 |  | 0.75456 | 0.12552 | **0.04433** | 0.28895 | 0.12418 | 0.87203 | 0.83726 | 0.63709 | 0.32305 | 0.48809 |

IPI, international prognostic index; PS, performance status; SA, surface area .Correlation was done using Spearman correlation. *P* <0.05 is statistically significant.

**Supplementary Table S2.Logistic regression analysis of plasma lncRNAs to predict DLBCL diagnosis**

| Variable | Univariate analysis | | | | |
| --- | --- | --- | --- | --- | --- |
|  | B-coeffecient | S.E. | *P* | OR | 95% CI |
| HOTAIR | 0.49294 | 0.472 | 0.2969 | 1.6362 | 0.6488-4.1266 |
| Linc-p21 | -1.6857 | 1.021 | 0.0987 | 0.1853 | 0.0251-1.3705 |
| GAS5 | -0.0328 | 0.063 | 0.6021 | 0.9677 | 0.8555-1.0947 |
| XIST | 1.3765 | 1.21 | 0.255 | 3.961 | 0.3694-42.4784 |

Univariate analysis was done in DLBCL (n=84) versus healthy controls (n=33).

*P* <0.05 is statistically significant.
